# Coenzyme Q10 Modulates Apoptotic Effects of Chronic Administration of Methadone on NMRI Mouse Hippocampus

**DOI:** 10.22074/cellj.2021.7384

**Published:** 2021-10-30

**Authors:** Maryam Vaselbehagh, Mehdi Sadegh, Hadi Karami, Saied Babaie, Mohammad Hassan Sakhaie

**Affiliations:** 1. Department of Anatomy, Faculty of Medicine, Arak University of Medical Sciences, Arak, Iran; 2. Department of Physiology, Faculty of Medicine, Arak University of Medical Sciences, Arak, Iran; 3. Department of Molecular Medicine and Biotechnology, Faculty of Medicine, Arak University of Medical Sciences, Arak, Iran

**Keywords:** Apoptosis, *Bdnf*, CoQ10, Hippocampus, Methadone

## Abstract

**Objective:**

Methadone is one of the widely used drug substances prescribed in treatment of opioid dependence and pain management; however, several studies have shown its neurotoxic effects on individuals and animal models. The purpose of this study was to assess neuroprotective effects of Coenzyme Q10 (CoQ10) on neurotoxicity induced by methadone in hippocampus of adult NMRI male mice.

**Materials and Methods:**

In this experimental study, 48 adult NMRI male mice were randomly divided into 4 groups (n=12 in each) including Methadone, Methadone with sesame oil, Methadone with CoQ10 and saline. The injections of methadone, saline and sesame oil were performed intraperitoneally for 20 days. 24 hours after last injection, half of the animals in each group (n=6) were randomly assessed for evaluating of spatial memory by radial maze. Following behavioral study, animals were sacrificed, and their brains were removed to evaluate pyknotic cells through histological assessment. The remaining were used to study the expression of *Arc, Bax, Bcl-2* and *Bdnf* genes.

**Results:**

Results of the present study showed that daily administration of methadone increased the number of pyknotic neurons in the CA1 hippocampus and altered the expression of *Bax, Bdnf, Arc* and *Bcl-2*. However, it did not alter spatial memory comparing to saline group. CoQ10 treatment significantly reduced the number of pyknotic cells and expression of *Bax, Bdnf, Arc* when compared to the vehicle group treated by sesame oil. However, the expression of Bcl-2 significantly increased as a result of CoQ10 treatment.

**Conclusion:**

CoQ10 reduced the neuronal damage caused by methadone in the hippocampus CA1.

## Introduction

Methadone is a synthetic opioid derivative prescribed for treatment of pain syndrome and opioid-related dependencies to avoid withdrawal symptoms (1). Despite the extensive therapeutic use of methadone, some studies have shown it has destructive and harmful effects on perception and cognition in individuals (2).

It’s been shown that, the expression of some genes is changed within the body as a result of drug abuse (3). *Bdnf* is a member of the neurotrophic factor family, which plays a role in regulating survival and differentiation of neurons in the central and peripheral nervous system (4). *Arc*, a member of the immediate-early gene (IEG) family, is another neuroplastic protein that plays a vital role in learning and memory-related process (5). *Arc* causes a series of changes in the pattern of neuronal activity relative to synaptic plasticity and thus, optimizes the storage of information in the nervous system (6). Long-term consumption of methadone increases the expression of pro-apoptotic proteins in the cerebral cortex and hippocampus (7, 8), leading to cell death activation through mitochondrial-mediated pathway, and ultimately dendritic atrophy, abnormal neurogenesis, and neurodegeneration (9). Members of *Bcl-2* family proteins have a crucial role in survival and cell death regulation, from which *Bax* is an important proapoptotic player that induces mitochondrial-mediated pathway of apoptosis via oligomerization and induction of mitochondrial membrane disruption (10). 

In neurodegenerative disease oxidative stress disrupts glutathione homeostasis and produces reactive oxygen species (ROS) (11). An excessive increase in ROS generation and a reduction in defensive antioxidants lead to oxidative damage to DNA, lipids and proteins and therefore leading to cell damage (12). Neuronal cells in CNS have a lower antioxidant capacity than glia, that makes them more susceptible to such injuries (13, 14) and hence increasing the antioxidant defense could provide neuroprotective effects. CoQ10 is a natural fatsoluble antioxidant that found in cellular organelles such as peroxisome, lysosomes, Golgi vesicles, and inner mitochondrial membrane (15, 16). It has been considered as a neuroprotective agent for treatment of neurodegenerative diseases. Q10 reduces the damage to hippocampal neurons (17), prevents nerve damage, and inhibits lipid peroxidation by reducing radical species production (18).

Because of the extensive use of methadone in the treatment of addiction and its negative effects on learning disruption and neuronal damage in the brain, the aim of this study was to evaluate the neuroprotective potential of Q10 as a complementary therapy in reducing methadone-induced neuronal damage.

## Materials and Methods

### Animals and treatment groups

All experimental procedures were approved by the Review Board and Ethics Committee of Arak University of Medical Sciences (IR.ARAKMU.REC.1395.318). Forty eight adult NMRI male mice (30-35 g) were obtained from Razi Institute (Karaj, Iran). Animals were housed in 12-hour light/dark cycles and water and food were freely available. Mice were assigned into 4 following groups (n=12 in each) and treatments were administrated for 20 consecutive days: i. Saline group, ii. Methadone group: received methadone 10 mg/kg (i.p) daily, iii. Methadone+sesame oil group; received methadone 10 mg/kg (i.p) following the injection of sesame oil 0.2 ml/day (i.p), and iv. Methadone+Q10 group: received methadone 10 mg/kg (i.p) following the injection of Q10 dissolved in sesame oil 10 mg/kg daily for 20 consecutive days.

Twenty four hours after the last injection, mice from each group were randomly divided into 2 subgroups: half of them were assigned for behavioral study, then they were sacrificed and their brains were processed for morphological studies of pyknotic cells. Other half were used for gene expression analyses of *Arc, Bax, Bcl-2* and *Bdnf*.

### Histological study

The animals were killed by cervical dislocation, the brains were removed and fixed overnight in formalin10%. After paraffin embedding, coronal sections with 5μm thickness were prepared from -1.5 to -2.5 mm post bregma (in accordance with the mouse brain atlas, 2004). Sections were then dehydrated in the ascending alcohols series, stained with cresyl violet and mounted on glass slides. The slides were examined under a light microscope using an x40 objective lens (BX51, Japan) and images were captured by a digital camera (Olympus, DP 11, Japan). The number of pyknotic cells in each CA1 hippocampus were counted in three random areas of each section and five sections were analyzed for each sample. Pyknotic cells were characterized by their darkly stained condensed chromatin, a smaller volume and light or absent cytoplasm.

### Spatial learning test with radial arm maze

For assessment of memory and learning task, a radiating eight black Plexiglas arms maze with a central round platform was used as previously described with few modifications (19). Briefly the diameter of arms was 50×15×15 cm and each of arms numbered from 1 to 8. There was a removable door in the entrance of every arm of maze and at the end of it a well for hiding of food bite. Various intra and extra maze visual cues were sited through the testing room to help the animal for spatial memory task. Trials were performed daily 9:00 to 12:00 am. Habituation phase was done one day before the training session in which each mouse was placed in the RAM for adjustment of apparatus environment.

During the training phase, the animal was placed to the central platform and allowed for 30 seconds to become familiar with the place. After that the entry of all doors were opened and animal was allowed to freely explore the maze for 5 minutes. Four fixed arms were baited, and the others arranged in the same configuration throughout the entire experiment. The final time of spatial memory experiment were calculated when the mouse visited all four baited arms once or after 5 minutes, since the start of the trial.

Training session was done daily, for 5 continuous days. Access to food was restricted two hours before each trial. Following each trial, the apparatus was cleaned with 40% ethanol to avoid of olfactory cues.

To evaluate spatial learning and memory, two types of errors were calculated: i. Working memory errors (WME): the number of entries into a baited arm during each trial session. ii. Reference memory errors (RME): the number of entries into a non- baited arm during each trial.

### Gene expression study

After decapitation, the hippocampus was dissected and stored at nitrogen -80˚C until use. Total RNA was extracted using RNA plus extraction kit (Sinaclon, Iran). Three microgram RNA was reverse transcribed using oligo-dT primer (Yekta Tajhiz Azma, Iran) and reverse transcriptase (Yekta Tajhiz Azma, Iran) based on manufacturer’s protocol. The reaction mixtures were incubated at 42˚C for 60 minutes and then inactivated at 70˚C for 10 minutes. Resulting cDNA was subjected to quantitative real-time polymerase chain reaction (q-PCR) by using SYBR1 Green Mix (Yekta Tajhiz Azma, Iran) on a Light Cycler 96 System (Roche Diagnostics GmbH, Germany). The following conditions were used for q-PCR: initial heating for 10 minutes at 95˚C, 45 cycles of amplification, each composed of 15 seconds at 95˚C, 60 seconds at the annealing temperature, and 60 seconds at 72˚C. The annealing temperatures were 55˚C, 53˚C, 53˚C, 53˚C and 54˚C for *β-actin, Arc, Bdnf, Bcl-2* and *Bax*, respectively. Reactions were performed in triplicates. *β-actin* was used as an endogenous control to minimize the effect of sample variations. The fold changes in gene expression were calculated using ΔΔCt method. Primer were designed using Allele ID 7 software (Table 1). 

**Table 1:** Designed primers for the gene expression study


Gene name	Primer sequence (5ˊ-3ˊ)	Amplicon size

*Arc*	F: ACGACACCAGGTCTCAAG	159
	R: GCACCTCCTCTTTGTAATCC	
*Bax*	F: CTGAGCTGACCTTGGAGC	413
	R: GACTCCAGCCACAAAGATG	
*Bcl-2*	F: CACCCCTGGCATCTTCTCCT	349
	R: GACTCCAGCCACAAAGATG	
*Bdnf*	F: CACCCCTGGCATCTTCTCCT	118
	R: GTTGACGCTCCCCACACACA	
*β-actin*	F: GCGCCCATGAAAGAAGTAAA	536
	R: GGGCCGCTCTAGGCACCAA	


### Statistical analysis

Data are presented as mean ± SEM. Statistical analyses were performed using GraphPad Prism V8.4.0 (GraphPad Software, San Diego, CA, USA). One-way analysis of variance (ANOVA) was used for histological, WME, RME and gene expression data, followed by Bonferroni post-hoc test for further pairwise comparisons. Latency of spatial learning was analyzed by repeated measures ANOVA followed by Bonferroni post-hoc test. P<0.05 was regarded as statistically significant.

### CoQ10 reduced the number of Methadone-induced pyknotic cells in CA1

**Fig.1:**
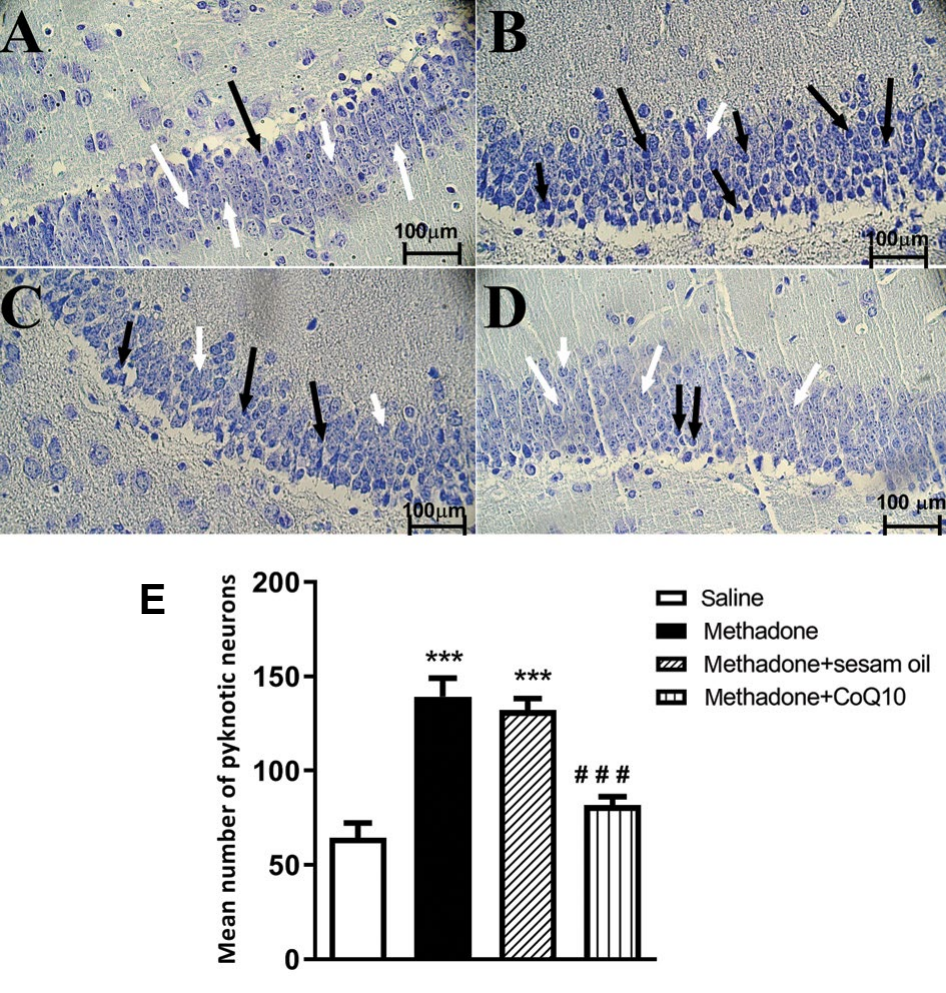
Nissl staining showed CoQ10 reduced the number of pyknotic cells in CA1 area. Statistical analysis demonstrated a significant increase of the pyknotic cells in methadone and methadone+sesame oil groups. CoQ10 significantly decreased pyknotic cells. Black arrows show pyknotic cells and white arrows show normal neurons. **A.** Saline, **B.** Methadone,** C. **Methadone+sesame oil, **D. **Methadone+CoQ10 group (scale bar: 100 µm). **E. **Mean values of the experimental groups. ***; P<0.001 compared with saline, ###; P<0.05 compared with methadone. All data are expressed as mean ± SEM.

As demonstrated in Figure 1, the numbers of pyknotic cells in CA1 significantly increased in the methadone group when compared to the control group (treated with saline) (P<0.001). Daily administration of CoQ10 to the mice treated with Methadone, significantly reduced the effect of methadone on pyknotic cells number (P<0.05). In addition, injection of Sesame oil without CoQ10 to the mice treated with Methadone showed no significant effects.

### CoQ10 did not alter the effects of methadone on spatial memory

**Fig.2:**
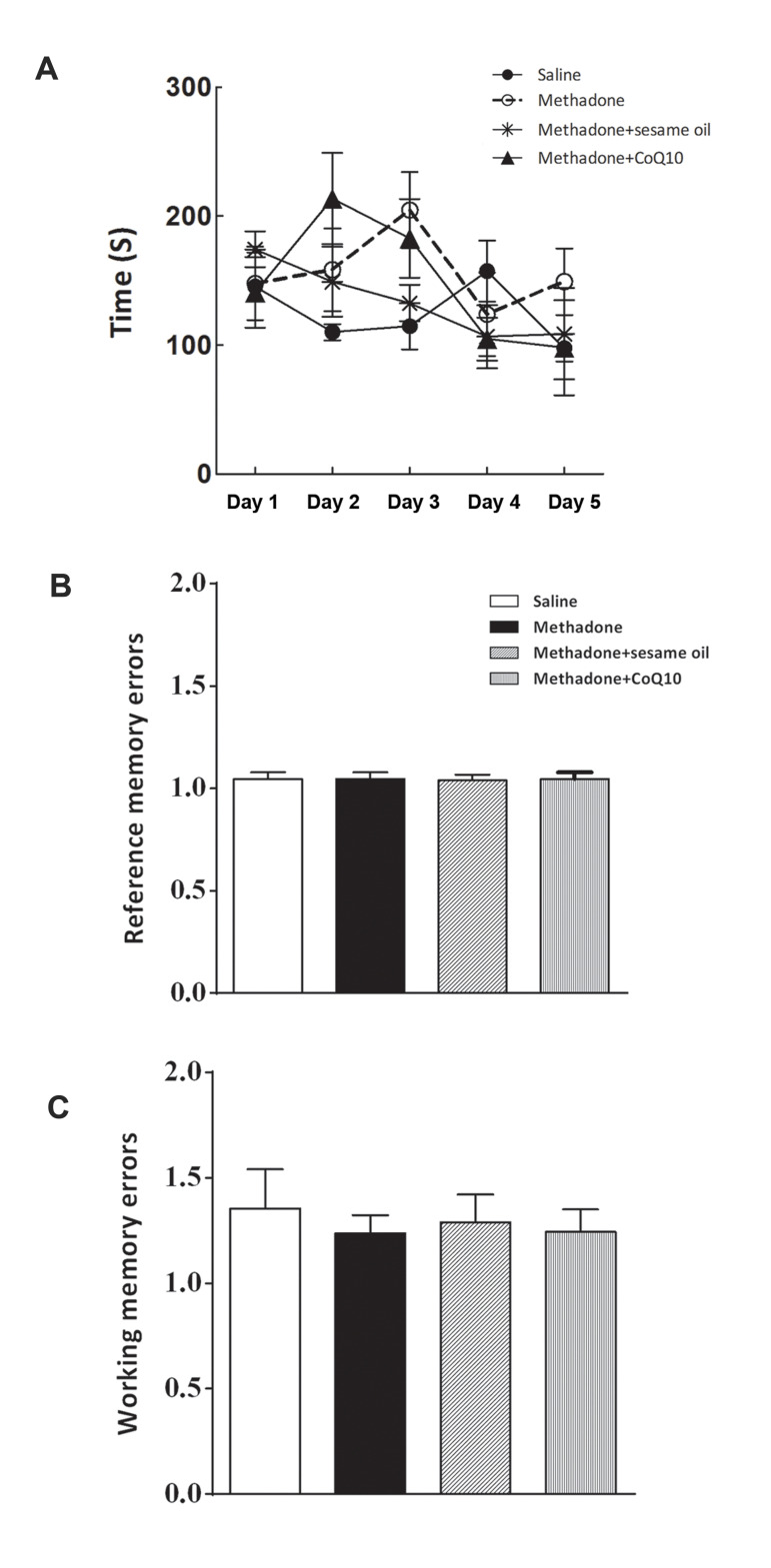
CoQ10 did not change the effects of methadone on spatial memory. **A. **Repeated measured ANOVA analysis showed no significant differences among experimental groups in latency. One-way ANOVA showed no significant differences among experimental groups in **B. **Working memory and C. Reference memory errors. The data are presented as mean ± SEM.

RMA analyses demonstrated there were no significant differences in mean values of latency to finish the four baited arms (F_12, 60_=1.4, Fig .2A) among experimental groups. Similarly, the mean number of entries into the baited arm during the trials (working memory errors, Fig .2B) and mean number of entries into a non -baited arm (reference memory errors, Fig .2C) remained unchanged.

### CoQ10 modified the effects of methadone on hippocampal gene expression

**Fig.3:**
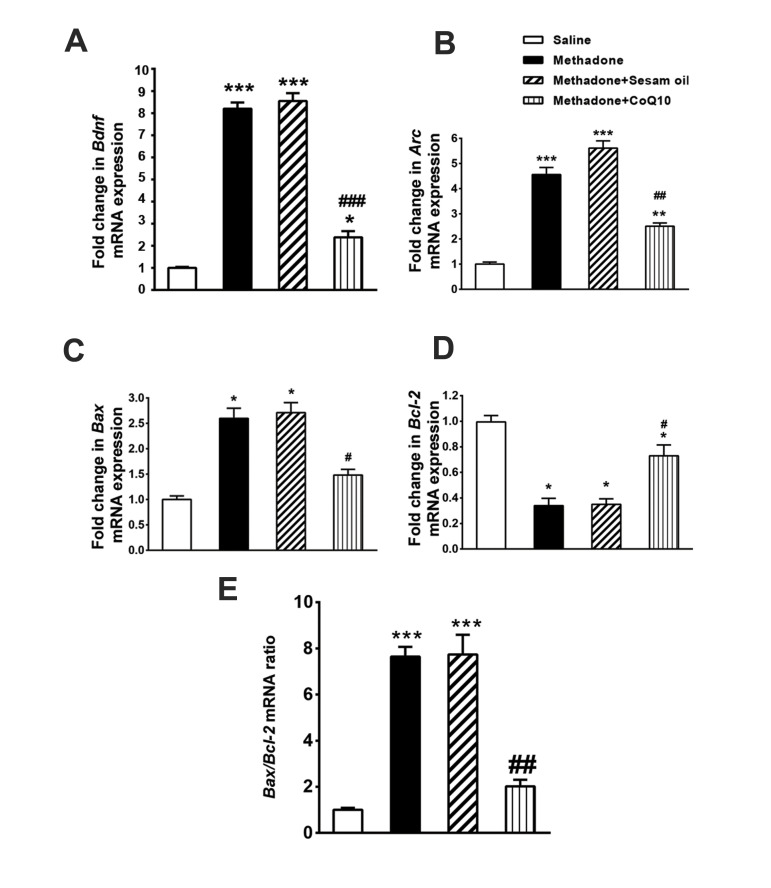
CoQ10 modified the effects of methadone on hippocampal gene expression. **A-C.** The relative expression of *Bdnf, Arc* and *Bax* in methadone group showed a significant increase compared to the saline group (P<0.001). **D. **However, relative expression of *Bcl-2* in methadone group showed a significant decrease compared to the saline group (P<0.01). **E.** In addition, *Bax/Bcl2* mRNA ratio significantly increased by methadone. Administration of CoQ10 following methadone significantly attenuated these effects. All data are expressed as mean ± SEM. *; P<0.05: relative to the saline group, **; P<0.01: relative to the saline group, ***; P<0.001: relative to the saline group, #; P<0.05: relative to methadone group, ##; P<0.01: relative to methadone group, and ###; P<0.001: relative to methadone group.

The results of quantitative PCR showed a significant enhancement in relative expression of *Bdnf* and *Arc* in all groups received methadone compared to the saline group (P<0.05). However, CoQ10 treatment significantly inhibited upregulation of both genes (P<0.05) in comparison with methadone group and methadone+ sesame oil group (Fig .3A, B). 

The relative expression of *Bcl-2* was significantly reduced in methadone group compared to the saline group (P<0.05), however; the relative expression of *Bax* was significantly increased (P<0.05). Treated animals with CoQ10 showed significant increase in *Bcl-2* expression compared to methadone group and methadone+sesame oil (P<0.05, Fig .3C). In addition, relative expression of *Bax* was significantly reduced in methadone+CoQ10 group in comparison with methadone group and methadone+sesame oil group (P<0.05, Fig .3D). Also, *Bax/Bcl-2* ratio demonstrate a significant increase in the methadone group and methadone+sesame oil group compared to Saline group while CoQ10 administration with methadone significantly reduced this effect (P<0.01, Fig .3E).

## Discussion

Results of the present study suggested that chronic administration of methadone led to an increase in number of pyknotic cells in CA1 area of hippocampus, while did not affect spatial memory assessed by radial maze analysis. In addition, the rate of mRNA expression of *Bax* gene increased, while the expression of anti-apoptotic gene *Bcl-2* declined. Methadone administration also increased the expression of the *Bdnf* and *Arc* genes. CoQ10 significantly reduced pyknotic cells and *Bax* gene expression, butincreased gene expression of *Bcl-2, Bdnf* and *Arc*.

Perez-Alvarez et al. (20) examined the effects of methadone on mitochondria in the SH-SY5Y cells and showed that high accumulation of methadone resulted in cell death.Therefore, the use of opiates may also lead to irreversible neuronal damage and death of neurons. In agreement with this result our histological finding indicated increased pyknotic cells in the CA1 hippocampus after methadone administration.

Friesen et al. (21) showed that methadone inhibited proliferation of myeloid leukemia cell line (HL-60) and induced cell death through caspase 3, 9 activation and diminishing of anti-apoptotic genes expression. Changes in calcium homeostasis in the presence of methadone can induce ROS production and finally cell death (20). Therefore, we aimed to evaluate the protective effects of coenzyme Q10 (CoQ10) against the adverse effects of methadone.

In the spatial memory test, our results showed no significant difference in the learning capacity of mice chronically treated with methadone when compared to the control group. This finding was consistent with the results of a study conducted by Cummins et al. (22) in which, chronic use of methadone showed no statistically significant difference in memory tests. 

In agreement with our findings Sadegh et al. (23) evaluated the effects of repeated injections of morphine on spatial memory and found no significant differences among experimental groups. However, they had shown hippocampus CA1 synaptic plasticity changed in morphine dependent rats due to chronic morphine consumption (24). Hence, it’s possible that methadone, similar to morphine, also affect the hippocampal neurons with no sign of behavioral learning.

Our histological findings showed increased number of pyknotic cells in the hippocampus CA1 of methadone administrated animals suggesting cell death induction. Previous molecular studies have shown chronic administration of morphine and other opiates deceased the number of hippocampal progenitor cells and newly BrdU positive cells in dentate gyrus (25, 26). Opiates might directly act on the progenitor cell population. In another study, TUNEL assay analysis indicated that chronic treatment of high dose morphine and it’s withdrawal, induced apoptotic cell death in the brain tissue (27). 

Interestingly, the results of our study indicated that CoQ10 treatment can reduce the number of pyknotic cells in hippocampal CA1 following hippocampal methadone injury. Natural antioxidants, such as CoQ10, are effective in improving mitochondrial complexes dysfunction and inhibition of oxidative stress damages. Hwang et al. (28) demonstrated that pretreatment of CoQ10 significantly prevented motor deterioration in a rat model of spinal cord ischemia-reperfusion damage, as a result of reduction in oxidative stress and neuronal apoptosis. Similarly, CoQ10 pretreatment could alleviate hippocampal neuronal loss and aberrant mossy fiber induced by kainic acid as a model of temporal lobe epilepsy referred to its potential to modulate the production of oxidative stress (29). Neuroprotective function of CoQ10 is associated with its scavenging capacity on free radicals (30). In our study methadone increased *Bdnf* gene expression in hippocampal tissue of methadone group compared to the saline group. In line with these results, Baydyuk proposed that *Bdnf* plays a vital role in maintenance and proper function of neuronal population. It also improves the survival of the immature striatal neurons and facilitates establishment of striatal connections during brain development. Furthermore it plays a crucial role in opioid-induced plasticity (31, 32). 

Analysis of a human study indicated that the serum levels of *Bdnf* increased in the methadone maintenance treatment patients compared to healthy controls (33). In alignment with our study Rouhani et al. showed morphine administration increased the expression of *Bdnf* gene in hippocampus of rats with a dose dependent manner (3).

The mechanism of the action of multiple protective effects of *Bdnf* against brain injury are related to its antiapoptosis, antiinflammation and antineurotoxicity effects (34). Therefore, the increased level of hippocampal *Bdnf* in our study could be a protective response triggering as a result of neuronal cell damages. Treatment with CoQ10 could reduce the level of *Bdnf* gene expression to almost the baseline.

The results of this study showed that methadone increased the expression of *Bax* and reduced the expression of *Bcl-2* when compared to the saline group. In consistent with our results, Tramullas et al. demonstrated that the chronic consumption of methadone and heroin in animals increased pro-apoptotic proteins and their activity in the cortex and hippocampus (7, 35). But after treatment with CoQ10, the expression of *Bax* decreased and the expression of *Bcl-2* increased. Similar to our study, Gholipour et al. (17) showed that CoQ10 could reduce apoptosis induced by methamphetamine through reducing apoptotic factors and increasing the anti-apoptotic pathways in the rat brain. Lee et al. (36) provided evidence that CoQ10 promotes survival in ischemic mouse retinal cells by inhibiting oxidative stress and blocking the *Bax/Bad* mediated mitochondrial apoptotic pathway. CoQ10 is also able to activate the phosphatidylinositol-3-kinases (PI3K) pathway in neurons and significantly reduces the amount of ROS production (37). As a limitation of this study we did not asses the ROS alterations in our experimental groups and it should be considered for the future studies.

In summary, for the first time our results showed methadone increased hippocampal gene expression of *Arc* and CoQ10 when administrated with methadone was able to prevent this effect. As an immediate early gene, *Arc* contributes to the synaptic plasticity and memory formation. Previous studies have shown that chronic morphine administration increased mRNA expression of immediate early genes such as *Arc* in the hippocampus (3, 38). Also, it has been shown that *Arc* is able to block cellular pathways associated with apoptosis (13). Therefore, it is possible that increased expression of *Arc* occurring following chronic methadone administration works as a protective feedback mechanism against the apoptotic effects of methadone. In addition, CoQ10 might preventthe expression of *Arc* gene by blocking the apoptotic effects of methadone.

## Conclusion

Finding of our study showed that CoQ10 reduced the neuronal damage and complications of methadone on hippocampus CA1, probably by modifying the expression of pro-apoptotic and anti-apoptotic genes. CoQ10 might be considered as complementary therapy to prevent adverse effects of methadone on hippocampus.

## References

[1] Sankararaman A, Masiulis I, Richardson DR, Andersen JM, Mørland J, Eisch AJ (2012). Methadone does not alter key parameters of adult hippocampal neurogenesis in the heroin-naïve rat. Neurosci Lett.

[2] Ahmad-Molaei L, Hassnian-Moghaddam H, Farnaghi F, Tomaz C, Haghparast A (2018). Delay-dependent impairments in memory and motor functions after acute methadone overdose in rats. Front Pharmacol.

[3] Rouhani F, Khodarahmi P, Naseh V (2019). NGF, BDNF and Arc mRNA expression in the hippocampus of rats after administration of morphine. Neurochem Res.

[4] Kowiański P, Lietzau G, Czuba E, Waśkow M, Steliga A, Moryś J (2018). BDNF: a key factor with multipotent impact on brain signaling and synaptic plasticity. Cell Mol Neurobiol.

[5] Gallo FT, Katche C, Morici JF, Medina JH, Weisstaub NV (2018). Immediate early genes, memory and psychiatric disorders: focus on c-Fos, Egr1 and Arc. Front Behav Neurosci.

[6] Bramham CR, Alme MN, Bittins M, Kuipers SD, Nair RR, Pai B (2010). The Arc of synaptic memory. Exp Brain Res.

[7] Tramullas M, Martínez-Cué C, Hurlé MA (2007). Chronic methadone treatment and repeated withdrawal impair cognition and increase the expression of apoptosis-related proteins in mouse brain. Psychopharmacology.

[8] Nouri F, Afarinesh MR, Sheibani V, Foroumadi A, Esmaeili Mahani S, Mahmoudi M (2019). Concomitant abuse of methadone and methamphetamine could impair spatial learning and memory in male rats. Learn Motiv.

[9] Wang Y, Li W, Li Q, Yang W, Zhu J, Wang W (2011). White matter impairment in heroin addicts undergoing methadone maintenance treatment and prolonged abstinence: a preliminary DTI study. Neurosci Lett.

[10] Renault Thibaud T, Floros Konstantinos V, Elkholi R, Corrigan KA, Kushnareva Y, Wieder Shira Y (2015). Mitochondrial shape governs bax-induced membrane permeabilization and apoptosis. Mol Cell.

[11] Johnson WM, Wilson-Delfosse AL, Mieyal J (2012). Dysregulation of glutathione homeostasis in neurodegenerative diseases. Nutrients.

[12] Salim S (2017). Oxidative stress and the central nervous system. J Pharmacol Exp Ther.

[13] Bell KFS, Al-Mubarak B, Martel MA, McKay S, Wheelan N, Hasel P (2015). Neuronal development is promoted by weakened intrinsic antioxidant defences due to epigenetic repression of Nrf2. Nat Commun.

[14] Fernandez-Fernandez S, Almeida A, Bolanos JP (2012). Antioxidant and bioenergetic coupling between neurons and astrocytes. Biochem J.

[15] Sikorska M, Lanthier P, Miller H, Beyers M, Sodja C, Zurakowski B (2014). Nanomicellar formulation of coenzyme Q10 (Ubisol-Q10) effectively blocks ongoing neurodegeneration in the mouse 1-methyl- 4-phenyl-1, 2, 3, 6-tetrahydropyridine model: potential use as an adjuvant treatment in Parkinson’s disease. Neurobiol Aging.

[16] Balakrishnan P, Lee BJ, Oh DH, Kim JO, Lee YI, Kim DD (2009). Enhanced oral bioavailability of Coenzyme Q10 by self-emulsifying drug delivery systems. Int J Pharm.

[17] Gholipour F, Shams J, Zahiroddin A (2017). Protective effect of coenzyme q10 on methamphetamine-induced apoptosis in adult male rats. Novel Biomed.

[18] Sakhaie MH, Soleimani M, Pirhajati V, Soleimani Asl S, Madjd Z, Mehdizadeh M (2016). Coenzyme Q10 ameliorates trimethyltin chloride neurotoxicity in experimental model of injury in dentate gyrus of hippocampus: a histopathological and behavioral study. Iran Red Crescent Med J.

[19] Schrott LM, Franklin LTM, Serrano PA (2008). Prenatal opiate exposure impairs radial arm maze performance and reduces levels of BDNF precursor following training. Brain Res.

[20] Perez-Alvarez S, Cuenca-Lopez MD, de Mera RMMF, Puerta E, Karachitos A, Bednarczyk P (2010). Methadone induces necrotic-like cell death in SH-SY5Y cells by an impairment of mitochondrial ATP synthesis. Biochim Biophys Acta.

[21] Friesen C, Roscher M, Alt A, Miltner E (2008). Methadone, commonly used as maintenance medication for outpatient treatment of opioid dependence, kills leukemia cells and overcomes chemoresistance. Cancer Res.

[22] Cummins E, Allen CP, Ricchetti A, Boughner E, Christenson K, Haines M (2012). The effects of acute and chronic steady state methadone on memory retrieval in rats. Psychopharmacology (Berl)..

[23] Sadegh M, Fathollahi Y, Naghdi N, Semnanian S (2013). Morphine deteriorates spatial memory in sodium salicylate treated rats. Eur J Pharmacol.

[24] Sadegh M, Fathollahi Y, Semnanian S (2013). The chronic treatment in vivo of salicylate or morphine alters excitatory effects of subsequent salicylate or morphine tests in vitro in hippocampus area CA1. Eur J Pharmacol.

[25] Zhang Y, Xu C, Zheng H, Loh HH, Law PY (2016). Morphine modulates adult neurogenesis and contextual memory by impeding the maturation of neural progenitors. PLoS One.

[26] Zhang Y, Loh HH, Law PY (2016). Effect of opioid on adult hippocampal neurogenesis.

[27] Emeterio EPS, Tramullas M, Hurlé MA (2006). Modulation of apoptosis in the mouse brain after morphine treatments and morphine withdrawal. J Neurosci Res.

[28] Hwang JY, Min SW, Jeon YT, Hwang JW, Park SH, Kim JH (2015). Effect of coenzyme Q10 on spinal cord ischemia-reperfusion injury. J Neurosurg Spine.

[29] Baluchnejadmojarad T, Roghani M (2013). Coenzyme q10 ameliorates neurodegeneration, mossy fiber sprouting, and oxidative stress in intrahippocampal kainate model of temporal lobe epilepsy in rat. J Mol Neurosci.

[30] Hargreaves IP (2014). Coenzyme Q10 as a therapy for mitochondrial disease. Int J Biochem Cell Biol.

[31] Baydyuk M, Xu B (2014). BDNF signaling and survival of striatal neurons.Front Cell Neurosci.

[32] Cowansage KK, LeDoux JE, Monfils MH (2010). Brain-derived neurotrophic factor: a dynamic gatekeeper of neural plasticity. Curr Mol Pharmacol.

[33] Tsai MC, Huang TL (2017). Brain-derived neurotrophic factor (BDNF) and oxidative stress in heroin-dependent male patients undergoing methadone maintenance treatment. Psychiatry Res.

[34] Chen A, Xiong LJ, Tong Y, Mao M (2013). The neuroprotective roles of BDNF in hypoxic ischemic brain injury. Biomed Rep.

[35] Tramullas M, Martíne z-Cué C, Hurlé MA (2008). Chronic administration of heroin to mice produces up-regulation of brain apoptosis-related proteins and impairs spatial learning and memory. Neuropharmacology.

[36] Lee D, Kim KY, Shim MS, Kim S, Ellisman MH, Weinreb RN (2014). Coenzyme Q10 ameliorates oxidative stress and prevents mitochondrial alteration in ischemic retinal injury. Apoptosis.

[37] Choi H, Park HH, Koh SH, Choi NY, Yu HJ, Park J (2012). Coenzyme Q10 protects against amyloid beta-induced neuronal cell death by inhibiting oxidative stress and activating the P13K pathway. Neurotoxicology.

[38] Yip KW, Reed JC (2008). Bcl-2 family proteins and cancer. Oncogene.

